# Cranial irradiation mediated spine loss is sex-specific and complement receptor-3 dependent in male mice

**DOI:** 10.1038/s41598-019-55366-6

**Published:** 2019-12-11

**Authors:** Joshua J. Hinkle, John A. Olschowka, Tanzy M. Love, Jacqueline P. Williams, M. Kerry O’Banion

**Affiliations:** 1 Department of Neuroscience and Del Monte Neuroscience Institute, University of Rochester School of Medicine & Dentistry, Rochester, New York 14642 USA; 2 Department of Biostatistics and Computational Biology, University of Rochester School of Medicine & Dentistry, Rochester, New York 14642 USA; 3 Department of Environmental Medicine, University of Rochester School of Medicine & Dentistry, Rochester, New York 14642 USA; 4 Department of Neurology, University of Rochester School of Medicine & Dentistry, Rochester, New York 14642 USA

**Keywords:** Microglia, Translational research

## Abstract

Cranial irradiation is the main therapeutic treatment for primary and metastatic malignancies in the brain. However, cranial radiation therapy produces long-term impairment in memory, information processing, and attention that contribute to a decline in quality of life. The hippocampal neural network is fundamental for proper storage and retrieval of episodic and spatial memories, suggesting that hippocampal signaling dysfunction could be responsible for the progressive memory deficits observed following irradiation. Previous rodent studies demonstrated that irradiation induces significant loss in dendritic spine number, alters spine morphology, and is associated with behavioral task deficits. Additionally, the literature suggests a common mechanism in which synaptic elimination via microglial-mediated phagocytosis is complement dependent and associated with cognitive impairment in aging as well as disease. We demonstrate sexual dimorphisms in irradiation-mediated alterations of microglia activation markers and dendritic spine density. Further, we find that the significant dendritic spine loss observed in male mice following irradiation is microglia complement receptor 3 (CR3)-dependent. By identifying sex-dependent cellular and molecular factors underlying irradiation-mediated spine loss, therapies can be developed to counteract irradiation-induced cognitive decline and improve patient quality of life.

## Introduction

The mechanisms underlying irradiation-mediated cognitive impairment are poorly understood. Depending on the irradiated brain region, cognitive dysfunction can manifest as alterations in executive function, attention, sensory perception, behavior, and memory, and significantly impact a person’s capacity for daily activities^1–3^. As anti-cancer therapies and cancer care improve, patients live longer, presenting new challenges as long-term side effects manifest^1,3^. For example, over 80% of adult tumor patients that received whole-brain radiation therapy and survived more than six months developed a form of cognitive dysfunction. In this group, 5% of survivors progressed from cognitive impairment to dementia requiring 24-hour care^2^. Currently, there are no treatments to prevent irradiation-mediated damage to normal brain tissue and subsequent long-term cognitive dysfunction. Part of the difficulty is we do not fully understand neuronal and glial changes, in particular, irradiation-mediated effects on neuronal structure and microglial activation that could impact cognitive function.

Microglia, the resident immune macrophages of the central nervous system (CNS), are intricately branched and respond rapidly to pathological changes in the brain parenchyma such as excitotoxicity, neurodegenerative insults, ischemia, and direct tissue damage^4,5^. Their response consists of actively moving towards the damage site, engulfing debris, and eliminating cell components following death. Recently, studies have revealed non-pathological functions for microglia, including regulation of synaptic and structural plasticity during learning and memory^6,7^. A possible candidate system linking microglial immunological functions and fine-tuned regulation of phagocytosis is the complement pathway.

Similar to other innate immune cells (monocytes and macrophages), microglia throughout the CNS constitutively express C1q and complement receptor-1, and upon activation, upregulate complement receptor-3 (CR3: CD11b/CD18; MAC-1) and are able to shed C3 protein. This upregulation and shedding enhance the complement cascade, leading to inflammation, phagocytosis of complement-tagged components, and immune reactions^8–10^. Complement component C3 is critical in all three-complement activation pathways (classical, alternative, and lectin) as it is the converging point for each pathway and provides protection against pathogens via C3 proteolytic cleavage into C3a, C3b, and iC3b fragments^8,11^. Complement signaling via the iC3b receptor, CR3, regulates a host of myeloid cell functions including chemotaxis, migration, adhesion, and phagocytosis of opsonized material^12^. Previous studies have shown that complement components C1q and C3 localize to synapses, facilitating microglia CR3-mediated phagocytosis and pruning during development^11,13,14^. Additional studies have shown that C3 deficiency leads to enhanced hippocampal-dependent learning, suggesting that C3-dependent phagocytosis hinders learning and memory in young and aging mice^15,16^. Other groups demonstrated that inhibition of C1q, C3, or CR3 rescued synaptic loss and cognitive impairment in a mouse model of Alzheimer’s disease^17,18^. Lastly, increased C1q and C3d deposits at synapses that localized within microglial processes were detected in post-mortem multiple sclerosis brain tissue^19^. Altogether, these results suggest a common mechanism whereby synaptic elimination via microglial-mediated phagocytosis is complement dependent and associated with cognitive impairment in aging and disease.

The dynamic regulation of active synapses is critical for efficient function of neuronal circuits as well as learning and memory following environmental and behavioral stimuli^20^. Since dendritic spines are responsible for neuronal connectivity and represent the primary recipients for excitatory input, changes in spine density and morphology (diameter and length) can cause changes in synaptic efficacy and overall, account for the functional differences related to learning and memory^21,22^. For instance, the spine head volume is directly proportional to the number of docked synaptic vesicles at the active zone, number of postsynaptic receptors, and the area of the postsynaptic density^22,23^. Additionally, abnormalities in spine morphology have been described in several conditions associated with cognitive decline; including, Alzheimer’s disease, Huntington disease, autism, and aging^24^.

Cranial irradiation is associated with tissue damage that likely accounts for neurocognitive complications that negatively impact patient quality of life. Following radiation exposure of a tumor, the surrounding healthy tissue is subjected to not only acute, but persistent, oxidative stress, reduced neurogenesis, neuroinflammation, and vascular changes, all of which have the potential to contribute to neurocognitive sequelae via decreased neuronal structural complexity and synaptic connections^21,23,25^. Recently, several groups have demonstrated changes in synaptic density and dendritic spine complexity following radiation exposure. For example, Parihar & Limoli showed decreased numbers of hippocampal dendritic branches and branch points, as well as reduced dendritic length and dendritic area following ^137^Cs doses of either 1 or 10 Gy at 30 days post cranial irradiation^26^. The number of spines, spine density, and filopodia/thin (‘learning’) spines were significantly reduced, indicating that irradiation has a robust effect on dendritic complexity and synaptic composition. Additionally, the authors showed a significant decrease in synaptophysin, a presynaptic marker implicated in novel object recognition and spatial learning^26^, further suggesting that radiation compromises neuronal connectivity and memory. Although some proton and heavy ion studies have failed to find radiation-associated cognitive deficits^27,28^, neuronal injury and cognitive changes can occur with these types of ionizing radiation and are therefore not specific to head-only gamma irradiation^29–31^. To date, the mechanisms underlying radiation-mediated loss of spine density and complexity are not fully understood. However, selective ablation of microglia using a colony stimulating factor-1 receptor (CSF1R) inhibitor abrogated irradiation-mediated deficits in hippocampal-dependent behavioral tasks^25,32–34^. Taken together, the literature implicates microglia as major effectors of synaptic change and raises the possibility that microglia are involved in radiation-induced neuronal damage and cognitive dysfunction.

It is increasingly recognized that an animal’s sex can significantly influence neural responses in development^35,36^, aging, disease^37,38^, and injury^39,40^. For example, microglia participate in developmental processes through estradiol-induced masculinization of dendritic spine patterning in male rodents, leading to structural alterations in neuronal circuitry and sex-specific behaviors^36^. Further, studies have demonstrated a distinct sex difference in regulated genes corresponding to inflammatory^40^, MHCI, and complement^37^ pathway components in the hippocampus, amygdala, hypothalamus, and preoptic area^37,41^. However, sex differences pre- and post-cranial irradiation are understudied in the literature and provide little data describing how female mice respond to radiation. Two recent studies using high-LET particle irradiation demonstrated sex differences in microglial activation, synaptic modifications, and cognitive deficits^42,43^, suggesting irradiation-mediated changes are dependent on sex.

To investigate the possible link between microglia and synaptic complexity following cranial radiation, we focused on the hippocampus; a critical structure involved in learning and memory, and investigated irradiation-mediated effects on microglial activation and neuronal structure in male and female mice. In particular, we used a genetic CR3 knockout strategy (deficiency in CD11b) to ask whether dendritic spine loss following irradiation is dependent on microglial CR3 expression. Our findings support a role for microglia in mediating radiation-associated changes in neuronal dendritic spine density that is CR3- and sex-dependent, suggesting the need for sex-specific novel therapeutic avenues to reduce radiation-mediated spine loss and improve patient quality of life.

## Results

**Figure 1:**
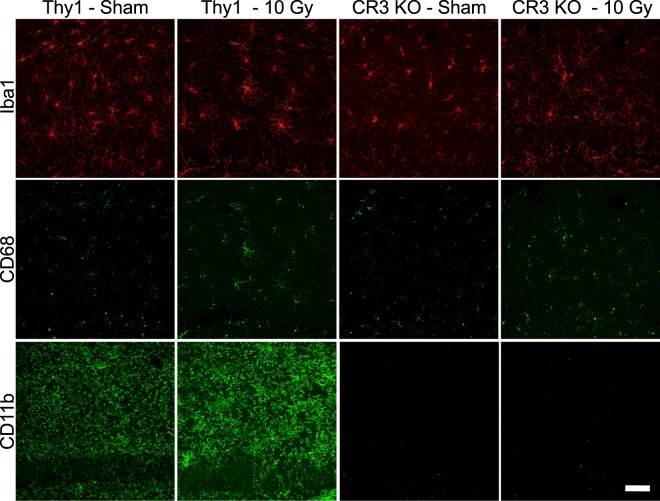
Representative confocal immunofluorescent max projection z-stack images from male Thy1+ and CR3 KO animals, either sham-irradiated or 10 Gy, taken in the upper portion of the hippocampal dentate gyrus molecular layer. Scale bar: 50 µm.

Classically, microglial activation has been characterized as a shift in morphology from a ramified arbor to a more amoeboid shape^44,45^. To investigate microglial morphology changes and activation after cranial radiation, sections from Thy1-eYFP (Thy1+) and CR3 KO male and female mice, subjected to sham-irradiation (sham) or a single 10 Gy γ-irradiation dose (IR), were analyzed at 30 d post-irradiation for microglial markers Iba1, CD68, and CD11b (representative male and female images - Fig. 1 and Supplemental Fig. S1, respectively). This dose and time point, as well as Thy1+ mice, were selected based on previous literature^21,26^ and all imaging and analyses were performed in the hippocampal dentate gyrus molecular layer (Supplemental Fig. S2). Further, in order to confirm that the Thy1+ genotype did not influence radiation responses, we carried out an additional experiment with wild-type C57BL/6 mice (WT) of the same age. We found no difference between these mice and the Thy1+ mice with regard to baseline or radiation-response for microglial markers or synaptic density (Supplemental Fig. S6 and Table S7).

### Irradiation does not induce changes in male microglial morphology; however, there is a significant basal sex difference in microglial morphology

**Figure 2:**
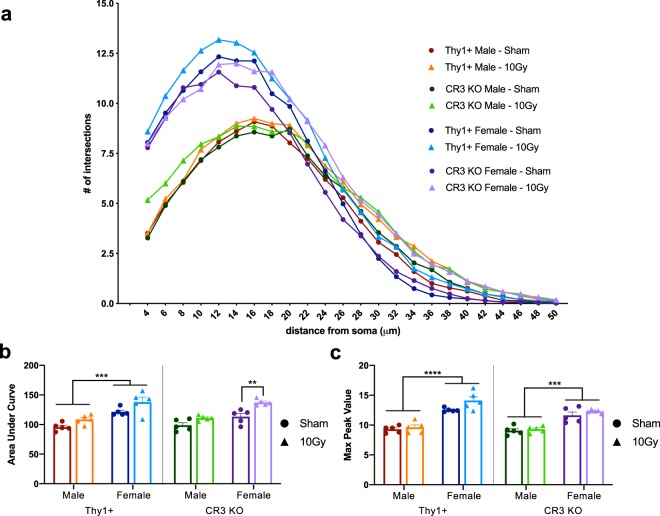
Morphological Sholl analysis of Iba1 stained microglial arbor showing (**a**) all eight groups together. (**b**) To further demonstrate differences, area under the curve and (**c)** max peak values were plotted demonstrating that irradiation did not have a significant effect on microglial morphology (except CR3 KO female mice); however, there was a significant sex difference in both Thy1+ and CR3 KO mice (Supplemental Fig. S3 and Table S5). n = 5 per group; (**b**,**c**) three-way ANOVA followed by multiple comparisons, *p < 0.05, **p < 0.01, ***p < 0.001, ****p < 0.0001.

Sholl analysis was performed on Iba1 stained sections to quantify the number of microglial process intersections as a function of radial distance from individual cell soma in the molecular layer of the hippocampus (Fig. 2a). Thy1+ and CR3 KO male mice showed no significant effect between IR and the distribution of microglia intersections (Fig. 2a – two-way ANOVA; Thy1: F_(23, 192)_ = 1.011, p = 0.453; CR3 KO: F_(23, 192)_ = 0.964, p = 0.515). Further, when comparing Thy1+ and CR3 KO male animals there were no significant differences in microglial arbor distribution (Fig. 2a), area under curve (Fig. 2b), max peak values (Fig. 2c), Iba1 immunoreactivity percent area covered, or microglial density (Supplemental Fig. S2; all three-way ANOVA data displayed in Supplemental Table S5). In the same cohort as the aforementioned males, quantification of female Thy1+ microglial morphology by Sholl analysis showed no significant effect while, interestingly, CR3 KO females showed a significant radiation effect that was most evident as increased distal process intersections, indicating lengthened distal branching (Fig. 2a – Thy1: F_(23, 168)_ = 0.304, p = 0.999; CR3 KO: F_(23, 144)_ = 2.42, p = 0.0008, *post hoc* multiple t-tests with Sidak corrections, 28–44 μm, p < 0.05). This increase in distal processes corresponded with a significant increase in area under curve and Iba1 percent area in CR3 KO females (Fig. 2b and Supplemental Fig. S2) while max peak values were not significantly different in control vs. irradiated female mice (Fig. 2c).

When comparing sham Thy1+ and CR3 KO mice, female mice had a significant increase in primary and medial process intersections, indicating a more complex inner branching while the number of distal processes were similar to male mice (Supplemental Fig. S3 – Thy1: F_(23, 168)_ = 30.19, p < 0.0001; *post hoc* 4–18 μm, p < 0.05; CR3 KO: F_(23, 192)_ = 20.07, p < 0.0001; *post hoc* 4–18 μm, p < 0.05). This basal increase in process intersections also corresponded with a significant increase in area under curve (Thy1+ only; Fig. 2b), max peak values (Fig. 2c), and Iba1 percent area covered (Supplemental Fig. S2) in female microglia when compared to male microglia while there was a non-significant trend towards decreased microglial density following irradiation in both male and female mice (Supplemental Fig. S1).

### Irradiation induces increases in CD68 and CD11b immunoreactivity in male mice and there is a basal sex difference in immunoreactivity levels

**Figure 3:**
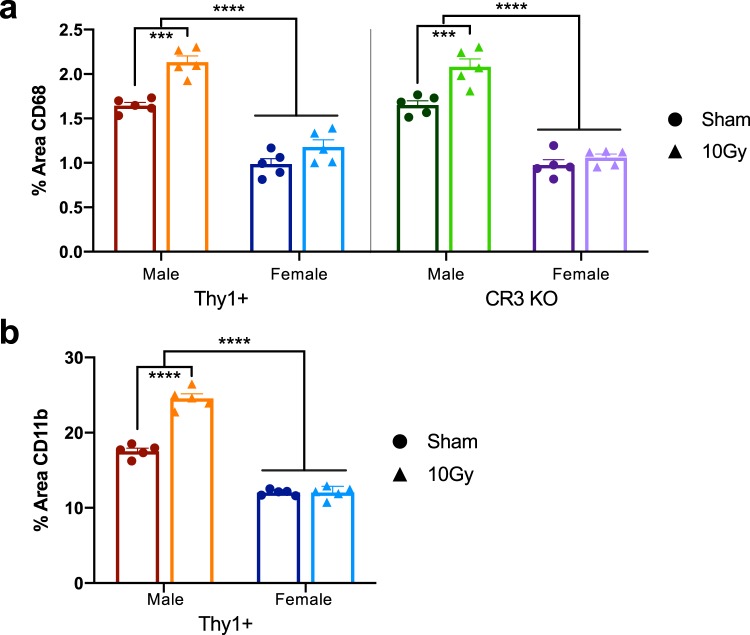
Quantification of microglial markers CD68 (**a**) and CD11b (**b**) immunofluorescence (percent area) were significantly increased in male Thy1+ and CR3 KO mice but unaltered in female mice following irradiation. Further comparison of CD68 and CD11b sham immunoreactivity showed a significant basal sex difference in Thy1+ and CR3 KO animals. n = 5 per group; (**a**) 3-way ANOVA with multiple comparisons, (**b**) 2-way ANOVA with multiple comparisons; ***p < 0.001, ****p < 0.0001.

In addition to morphological changes, internal microglial markers are modulated in response to changes in the microenvironment. As resident CNS macrophages, changes in phagocytic machinery represent a key component in injury response associated with enhanced cellular debris clearance and dendritic spine removal^9,15,46^. To demonstrate changes in microglial reactivity, more specifically, phagocytic machinery and CR3 levels, we used CD68 and CD11b, respectively. Following irradiation, percent area of CD68 was significantly increased in both Thy1+ and CR3 KO male mice (Fig. 3a – Thy1: p < 0.0001, CR3 KO: p = 0.0004). In addition, percent area of CD11b was significantly increased in Thy1+ male mice and, as expected, absent in CR3 KO animals (Fig. 3b – Thy1: p < 0.0001). Interestingly, irradiation did not alter CD68 or CD11b percent area in female animals and when comparing sexes, male sham mice had significantly higher expression of CD68 and CD11b immunoreactivity than female sham mice (Fig. 3 – p < 0.0001; Supplemental Table S4 – three-way ANOVA values; Supplemental Table S5 – mean ± SEM values).

### Immature spine density is significantly decreased following irradiation in male Thy1+ mice, but not in male CR3 KO or female mice

**Figure 4:**
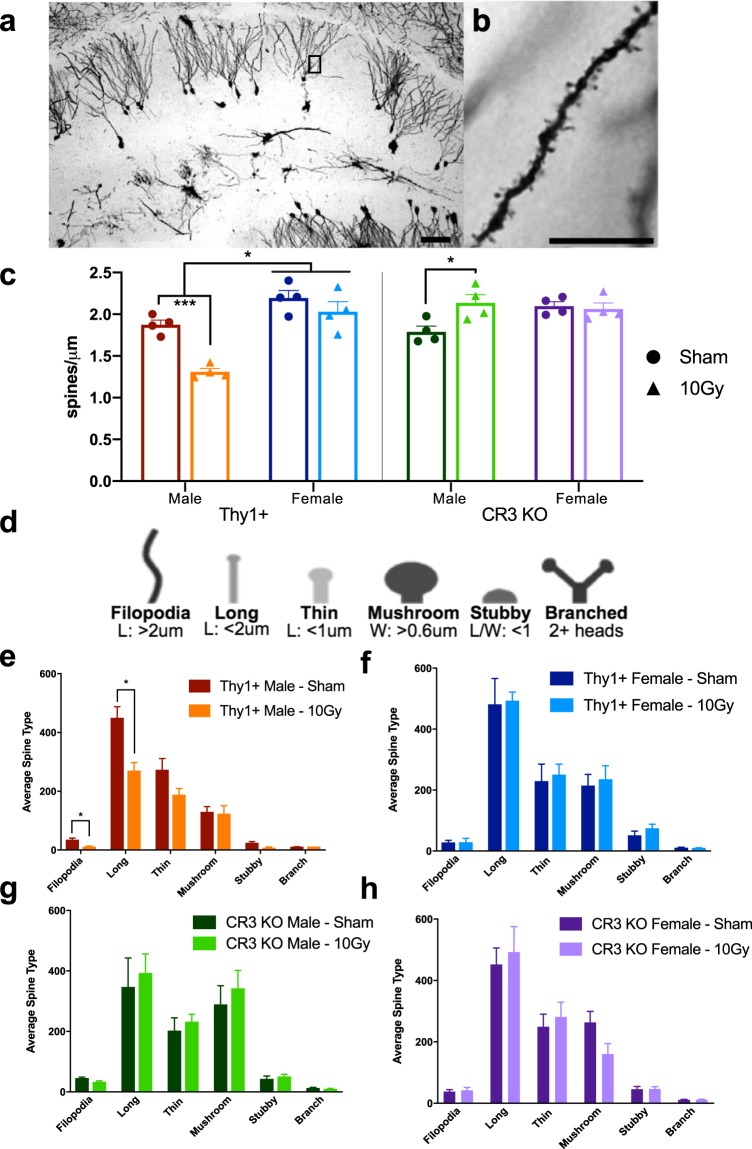
Quantification of dendritic spine density and morphology analysis. (**a**) Representative bright field image of Golgi stained hippocampus with high magnification inset showing spine protrusions (**b**). (**c**) Quantification of spine density (spines/μm) demonstrating a significant loss of spines in male Thy1+ mice but not CR3 KO male mice following irradiation while female spine density was unaltered in both Thy1+ and CR3 KO mice. (**d**) Schematic dictating spine category (adapted from^47^). (**e**–**h**) Golgi categorical quantification of spine morphology analysis showing the average spine type across all six categories in Thy1+ (**e**,**g**) and CR3 KO (**f**,**h**) sham and irradiated animals. n = 4 per group; (**c**) 3-way ANOVA with multiple comparisons, (**e**–**h**); multiple t-tests with Holm-Sidak correction (**e**,**g**). Scale bars: (**a**) 50 µm, (**b**) 10 µm.

To further investigate irradiation-mediated effects, Golgi-Cox stain was performed to measure spine density and potential changes in spine morphology following irradiation in the hippocampal molecular layer (Fig. 4). Dendritic spine subclasses were classified as filopodia, long, thin, stubby, mushroom, or branched, based on morphometric criteria^47^ (*Methods;* Fig. 4d). Thy1+ male spine density was significantly reduced after radiation exposure with filopodia and long spines being susceptible to radiation whereas more mature spine types were not affected 30 d post-irradiation (Fig. 4c – spine density: p = 0.0002; Fig. 4e – filo: p = 0.041, long: p = 0.042). In contrast, male CR3 KO mice showed a significant increase in spine density and no irradiation-associated change in spine types indicating CR3 plays a role in irradiation-mediated spine loss (Fig. 4c – spine density: p = 0.027; Fig. 4f – spine morphology: p > 0.05). Female mice did not demonstrate irradiation-mediated loss in spine density or distribution of morphology, indicating a lack of spine susceptibility in female mice (Fig. 4; p > 0.05). However, when comparing sexes, sham male mice demonstrated lower spine density than female mice indicating a sex difference in basal spine density in the molecular layer of dentate gyrus (Fig. 4c –Thy1: p = 0.039; CR3 KO: trend of p = 0.089; Supplemental Table S4 – three-way ANOVA values; Supplemental Table S5 – mean ± SEM values).

## Discussion

In this study, we demonstrate that microglia play a role in radiation-mediated synaptic loss and identify CR3-dependent signaling as the underlying mechanism in male mice. More specifically, we demonstrate in the hippocampal molecular layer 30 d post-irradiation that: i) there was little change in microglia morphology; ii) CD68 and CD11b immunoreactivity was up-regulated in male Thy1+ and CR3-KO mice; iii) there was a significant loss of spine density in male Thy1+ mice with enhanced vulnerability of immature spine populations; and iv) spine loss did not occur in CR3-KO male mice. Interestingly, these changes were limited to male mice as measures of CD68, CD11b (absent in CR3 KO), and spine density were unaltered by radiation in female mice and, indeed, basal conditions were significantly different compared to male mice.

### Cranial irradiation leads to elevation in microglial activation markers in male mice but not overt morphological changes

As microglia respond to injury, their activation state is reflected by a morphological shift from ramified and intricately branched cell bodies to a more amoeboid (rounded) shape that can be visualized through Iba1 immunohistological staining. By quantifying this transformation, or states of activation, analyses can reveal a spatiotemporal relationship between microglia morphology and the evolving injury^44,48^. Using Sholl analysis, which is well suited to analyze individual cell morphology and display topographical information^48^, we found no significant reduction in the number of intersections as a function of distance from the cell soma in Thy1+ or CR3 KO mice following irradiation. Interestingly, CR3 KO female mice showed a significant increase in intersections following irradiation, demonstrating a hyper-ramified phenotype that is not characteristic of increased inflammation. These data demonstrate that an acute dose of 10 Gy is not sufficient to elicit a sustained morphological response 30 days post-irradiation. A limitation to this observation is that we averaged changes across multiple microglia, potentially hiding a heterogeneous response to radiation with some cells showing more processes and others less. Moreover, this is a static snapshot of a dynamic process and other time points or higher radiation doses may demonstrate a difference.

Another important component of microglial activation is the ability to rapidly modify expression of cellular markers in response to changes in the microenvironment^46,48^. Two classical markers of microglial activity are CD68, a scavenger receptor that is predominately expressed on late endosomes and lysosomes^4,49^ and CD11b, an alpha M integrin that is integral in CR3 (CD11b/CD18 complex) mediation of extracellular membrane adherence and phagocytosis of complement-coated particles^8,50^. Although Iba1, CD68, and CD11b are not microglia-specific but also detect macrophages, the literature indicates little infiltration at 30 d following 10 Gy irradiation^51,52^ suggesting that macrophage infiltration is low (or transient) and should have a minimal impact on the microglial markers measured here. Our findings that CD68 and CD11b immunoreactivity are upregulated by radiation in Thy1+ male mice suggests the potential for increased CR3-mediated phagocytosis of opsonized material that is lacking in CR3 KO mice. CR3 KO mice show similar increased levels of CD68 immunoreactivity following irradiation, suggesting these microglia have an increased capacity for phagocytosis, though they are not able to do so via CR3-mediated removal. While our results are most consistent with microglial CR3-dependent phagocytosis of spines, future studies utilizing microglial specific CR3 deletions and direct measures of spine engulfment and phagocytosis will be required to test microglial CR3 specificty and dependence.

### Cranial irradiation leads to a significant loss in spine density, specifically immature spines

Damage to the hippocampal region has been associated with reduced spatial learning and impaired adaptability to behavioral tasks involving complex patterns and spatial pattern separation^3,15^. Hippocampal memory storage and retrieval occurs through the precise and simultaneous regulation of the dendritic arbor and the remodeling (growth or retraction) of synapses to properly code an experience-dependent memory. Since dendritic spines are responsible for neuronal connectivity and excitatory input, changes in spine density and morphology (diameter and length) could lead to changes in synaptic efficacy and overall account for the functional differences related to learning and memory^21,22,24^.

Several studies have demonstrated changes in spine density and dendritic complexity following radiation exposure. For example, low dose ^56^Fe particles (0.5 Gy, 600 MeV/n) altered hippocampal spine density and dendritic morphology in a region-specific manner, suggesting differential vulnerability in CA1, CA3, and DG regions^29,31^, while low dose ^16^O and ^48^Ti particles (0.05 and 0.3 Gy, 600 MeV/n) reduced dendritic complexity and spine density in the medial prefrontal cortex^30^. Further, a single dose of low LET 10 Gy gamma rays (head-only, ^137^Cs) showed persistent spine loss in the hippocampal dentate gyrus, but not in CA1^21^ and a sustained loss in dendritic complexity, spine density, and spine number in the hippocampal molecular layer^26^. In addition to spine loss, spine morphology can impact synaptic stability and strength by providing a malleable surface for receptor trafficking and calcium dynamics. The size of the spine head directly correlates with the number of a-amino-3-hydroxy-5-methyl-4-isoxazolepropionic acid (AMPA) receptors that can anchor, allowing an increase in post-synaptic excitability and synaptic strength^29,53^. Mushroom spines are thought to be mature and represent stable “memory” while filopodia and long spines, containing little to no AMPA receptors, are highly motile and considered to aid in synaptogenesis and plasticity^54^.

Previous radiation studies demonstrated increased vulnerability of “immature” (filopodia and long) spines when compared to more “mature” mushroom spines following radiation, and correlated spine loss with behavioral deficits and cognitive impairment in hippocampal-dependent tasks^26,30,31^. Since immature spines provide circuit flexibility and the potential to seek and accommodate new inputs, the authors concluded that decreasing this spine population could decrease the ability to form new synapses and alter neuronal activity in hippocampal-dependent learning tasks. While our studies do not provide a behavioral correlate, our results corroborate previous findings and demonstrate a 30.1% loss in spine density in Thy1+ male mice following irradiation with increased susceptibility in filopodia and long spines (68.1% and 39.9% reduction, respectively), but no change in mushroom spine numbers. Two previous studies demonstrated a reduction in spine density 30 d post-irradiation (10 Gy) in the hippocampus^21,26^; our spine density results are comparable to Charkraborti *et al*. while our spine morphology data are more similar to Parihar & Limoli. The Golgi-staining method, while sensitive and reliable, does not assess dendrites in three dimensions and therefore underestimates the overall spine number. This may account for the lesser extent of loss and overall decrease in total spine numbers when compared to Parihar & Limoli’s results. Further, our results showed significant reduction in immature spines and no changes in mushroom or stubby types, which contradicts Chakraborti *et al*., but is similar to Parihar & Limoli. One major difference was the generation of our spine classification, which was based on unbiased measurements of spine head and neck length^47^, whereas the former study used manual classification based on visual appearance. In addition, alterations in spine classification criteria and variations in sampling area could account for the differences in results.

### Irradiation-mediated spine loss is prevented in CR3-KO male mice

Microglia have been shown to be involved in experience-dependent plasticity through the modification and maintenance of synaptic elements in the adult brain^6,7,55,56^. These functions require local, fine-tuned signaling that involves the delivery of specialized targeted messages from individual synapses to microglial processes. This ultimately suggests that neuronal activity, neurotransmission, and sensory experience can modulate microglial process motility and physical contact between synapse and microglia. Previous work has provided evidence to support this hypothesis and has shown under homeostatic conditions that neuronal activity regulates microglial motility and process interactions with synaptic elements leading to functional plasticity^7,23,55–57^. The precise mechanism(s) linking microglia and synaptogenesis remains undefined; however, the complement system has been implicated in microglia-mediated synaptic removal in development^11^, aging^14^, and disease^4,17,19^.

One of the primary functions of macrophages is the recognition of tagged components and subsequent phagocytosis and removal. This function is facilitated by opsonization and occurs when C1q binds its target triggering a protease cascade and elevation of downstream complement C3 and C3 fragments (C3a, C3b and iC3b). Microglial CR3 recognizes iC3b, internalizes the target, and eliminates it via phagocytosis^8,11,17^. Previous studies have shown that complement disruption during development via C3 global KO resulted in delayed synaptic elimination in the hippocampus^58^ and sustained defects in synaptic connectivity and structural remodeling that were microglia-dependent^13^. C1q and C3 have been shown to be upregulated in aging with the most pronounced increase in the hippocampus^14^ and upon C3 deletion mice were protected against age-dependent synapse loss and had enhanced spatial learning and memory^15,16^. Complement has also been implicated in multiple sclerosis^19^ and Alzheimer’s disease^17^ as elevated C1q and C3 levels were correlated with increased microglial-dependent synaptic engulfment and synaptic loss, and consequent to C3 and CR3 depletion, microglial engulfment was significantly reduced. Few studies have investigated the role of complement and irradiation-mediated injury in the brain. One study showed C3 upregulation and accumulation along blood vessels (20 Gy)^59^ while another demonstrated that C3 deficiency was associated with increased learning following irradiation (8 Gy)^60^. However, C3 is not specific to any one cell type and is the converging point of all three complement pathways, so a deficiency may have widespread or ambiguous results.

In this study, our results demonstrate that CR3 deficiency provides dendritic spine protection and rescues the reduction observed in Thy1+ male mice following cranial irradiation. Due to elevated CD11b immunoreactivity in Thy1+ male mice and the specificity of CD11b to microglia in the brain, we suggest the spine loss observed in Thy1+ animals is facilitated by CR3-mediated phagocytosis. In addition, immature spine type vulnerability is not apparent in CR3 KO male mice and interestingly, CR3 KO irradiated spine density was elevated (19.4%) when compared to sham-irradiated CR3 KO controls. Although speculative, this increase could be due to the irradiation-mediated increase in microglial-specific extracellular matrix remodeling factors^61^ and the lack of CR3-mediated spine removal. Whether this irradiation-mediated increase in spine density in CR3 KO males and the loss of spines in Thy1+ correlates with altered hippocampal-dependent learning is an important question to be addressed in future studies. Although we are treating CR3 deficiency as a microglial specific knockout in the brain, a limitation is that the germline loss of CR3 will have wide-ranging effects during development and in the periphery. While the functional consequences between genotypes is difficult to predict and may influence radiation responses, our quantitative analyses demonstrate that CR3 KO animals show similar baseline levels for measures of microglial response, spine density, and spine morphology when compared to Thy1+ animals. Additionally, Thy1+ and WT mice exhibited similar levels of microglial markers and spine density measures suggesting that baseline levels and irradiation-responses are similar when comparing these two genotypes (Supplemental Figs. S6 and S7).

### Irradiation-mediated effects are sex dependent

A recent study demonstrated sexual dimorphisms in microglia gene expression under normal physiological conditions in young adult mice (3 mo) with the majority of differentially expressed male genes being associated with inflammatory processes; specifically, pro-inflammatory cytokine production and activity driven by transcription factor nuclear factor κB and the release of reactive oxygen species^39,62^. In contrast, differentially expressed female microglia genes had no association with inflammation and instead had profiles associated with development, cytoskeleton organization, and anti-inflammatory transcription factor activity. Further, the authors demonstrated that female microglia are more adept at reducing ischemic damage by transplanting female microglia into male brains depleted of microglia by CSF1R inhibition^39^. Another study demonstrated that male microglia were more reactive to LPS treatment while female microglia were unaffected, suggesting male mice have a more pro-inflammatory immune response^35^. Similarly, our results demonstrate a sex-specific difference in the inflammatory profile under physiological conditions as well as in response to irradiation: 1) female baseline levels of CD68 (Thy1+: −66.8%; CR3 −59.9%) and CD11b (Thy1+: −45.9%) immunoreactivity where significantly decreased when compared to males, suggesting reduced capacity for CR3-mediated phagocytosis at baseline; 2) female CD68 and CD11b percent area covered did not change following irradiation; 3) female microglial morphological complexity was significantly higher, as they had increased ramified primary and medial processes when compared to the less ramified, reactive male microglia; 4) interestingly, only female CR3 KO mice showed an irradiation-mediated morphological effect by displaying a significant increase in distal processes. These data suggest that microglial sexual dimorphisms may not be limited to contrasting inflammatory profiles demonstrated in the literature, but also reflect complement-dependent activity that is necessary for the proper regulation of dendritic spines. A limitation of our study is we did not monitor the estrous cycle at the time of irradiation and allowed the females to cycle randomly.

In addition to microglial sex differences, sexual dimorphisms in neuronal structure are also evident. There was no change in spine density following irradiation in female mice, and basal spine density was significantly elevated in Thy1+ (+14.7%) and CR3 KO (+14.6%) female mice when compared to male mice. This baseline elevation in spine density and the comparable densities in both Thy1+, CR3 KO, and WT female mice demonstrate a sex-specific variance under physiological conditions that is consistant with the literature^63^. Additionally, female spine density and spine morphology was unaltered by irradiation and had analogous densities to sham-irradiated females, suggesting that female mice either do not experience CR3-mediated spine loss following irradiation or are able to respond and recover from injury more efficiently than male mice. A recent study using high-LET particle irradiation (modeling galactic cosmic radiation; protons (60%; 252 MeV/n), helium (20%; 249.3 MeV/n), oxygen (20%; 594.4 MeV/n)) also demonstrated similar sex-specific changes in microglial activation and synaptic loss^42^, corroborating our findings and suggesting similar neuronal and microglial injury profiles across different radiation types. Together these results demonstrate sexual dimorphisms in both basal regulation of spine density and pathological response to radiation injury.

## Conclusion

The dynamic regulation of active synapses is critical for the efficient function of neuronal circuits as well as learning and memory following environmental and behavioral stimuli. Due to the detrimental effects of radiation on healthy tissue and the neurocognitive complications that follow, our study focused on the hippocampus, a region critical for learning and memory. Our findings provide insight into microglial and neuronal interactions and suggest a mechanism connecting irradiation-mediated spine loss and the complement pathway – specifically, microglial CR3-dependent phagocytosis. Additionally, our study emphasizes sexual dimorphisms in irradiation susceptibility as well as basal differences in spine density modulation and microglial phenotype. While there are many factors to be addressed, including behavioral endpoints, age, radiation dose, acute vs. fractionated doses, and the duration post-irradiation, our study provides a basis for sex-specific therapeutic approaches to mitigate radiation-associated changes in the neural network that may influence patient quality of life.

## Methods

### Animals, γ-irradiation, and tissue preparation

All animal procedures were carried out with ethical standards recommended by the Panel on Euthanasia of the American Veterinary Medical Association and approved by the University of Rochester Institutional Animal Care and Use Committee. *Thy1-eYFP* (Thy1+) transgenic mice (strain Tg(Thy1-YFP)HJrs, stock no. 003782, Jackson Laboratory, CT, USA), CR3-KO transgenic mice (strain B6.129S4-Itgam^tm1Myd/J^, stock no. 003991, Jackson Laboratory, CT, USA; a mutation in the CD11b gene resulting in a functionally deficient CR3 heterodimer), and C57BL/6 J (WT) mice (stock no. 000664, Jackson Laboratory, CT, USA) were housed with same-sex littermates in an approved and carefully monitored facility in the University Vivarium under the direction of trained veterinary staff. Optimal conditions were provided including adequate ventilation and temperature and light control; 12:12-hr light:dark cycle. Food and water were supplied ad libitum. Mice were generated from established in-house colonies (Thy1 breeding pairs generously provided by Ania Majewska) and genotyped to confirm the *Thy1* or *CR3* transgene. *Thy1*^*YFP/YFP*^*, CR3*^−/−^, or C57BL/6J male and female mice were used for all experiments. Female animals were allowed to cycle naturally and were not selected for estrous cycle synchronization.

Two month old mice were anesthetized (i.p. injection of ketamine [100 mg/kg] and xylazine [10 mg/kg]) and placed supine on a ^137^Cs irradiator (J. L. Shepherd and Associates, San Fernando, CA) with brain volume (between eyes and ears) positioned over a 5 mm × 12.2 cm collimator slit to provide a uniform field at a dose rate of 1.17 Gy/min for a total exposure of 10 Gy γ-irradiation. The collimator provided a uniform field with sharp edges that fell to a dose rate of 0 Gy/min, 2.5 mm from the slit edge^51^, providing a controlled area of cranial irradiation. Following radiation all animals were given a code number and the experimenter was blind in all subsequent analyses.

Thy1+, CR3-KO, and WT animals, 5 males and 5 females across two groups, sham-irradiated and irradiated, were sacrificed 30 d post-irradiation via transcardial perfusion with saline. Brains were removed and hemisected for immunohistochemical (IHC) analysis and Golgi staining procedure. Tissue for IHC was placed in 4% paraformaldehyde for 24 h, submerged in 30% sucrose for 48 h, flash frozen, and sectioned coronally (50 μm) via a freezing stage microtome (Microm HM400). Tissue for Golgi stain analysis was processed according to FD Rapid GolgiStain kit protocol (FD NeuroTechnologies). Following impregnation of tissue in solution A:B (14 d) and subsequently solution C (3 d), the tissue was flash frozen using dry-ice chilled isopentane and stored in −80 °C until sectioning. The tissue was coronally sectioned using a freezing stage microtome (100 μm thick). Sections were then freely floated in solution C, mounted onto gelatin-coated slides, and stored in the dark to dry for 24 to 72 h. Slides were then stained according to FD Rapid GolgiStain instructions (“VI:Staining Procedure”) and coverslipped using DPX (Electron Microscopy Sciences, #13510).

### Immunohistochemistry

Brain sections were washed (4 × 3 min in 0.15 M phosphate buffer (PB)), blocked in a PB solution of 6% normal goat serum (NGS) and 0.4% Triton-X for 1 h, then placed in a primary antibody PB solution containing 0.4% Triton-X and 3% NGS for 48 h at 4 °C. Primary antibodies consisted of Iba1 (Rabbit; Wako 019-19741; 1:2000) paired with CD68 (Rat; Bio-Rad MCA1957; 1:1000) or CD11b (Rat; Bio-Rad MCA74G; 1:1000). Following primary antibody incubation, sections were washed (4 × 3 min in PB), placed in a fluorescent secondary antibody PB solution of 0.4% Triton-X and 3% NGS for 2 h, removed from secondary, washed in PB (4 × 3 min), stained with Hoechst stain (Sigma-Aldrich, H6024 23491-45-4; 1:100) for 7 min, washed in PB (4 × 3 min), mounted on gelatin-coated slides, and coverslipped with Prolong Gold (Invitrogen, P36930). Secondary antibodies consisted of Alexa-Fluor Goat x Rabbit 647 (Invitrogen, A21245) and Goat x Rat 594 (Invitrogen, A11007) diluted at 1:2000. All steps were done at room temperature unless noted.

### Imaging

Following immunostaining, coronal sections containing the anterior portion of the dorsal hippocampus (−2.0 to −2.5 mm from Bregma) were imaged using an Olympus FV-1000 Confocal Microscope with a SIM 2-laser synchronized scanner (Olympus, Tokyo, Japan). Using Hoechst stain as a reference and framing, four z-stacks per animal were taken across the overlying molecular layer of the dentate gyrus with comparable sections selected across animals. Each image contained the stratum lacunosum moleculare as a top boundary and dentate gyrus granule cell layer as a bottom boundary (Supplemental Fig. S2, red box). Each z-stack was 20 μm thick (1024 × 1024 pixels) and acquired at 40x with 0.5 μm steps (Olympus UPLFLN 40x/1,30 Oil) for a total of 40 images per stack. Each image contained Iba1-647 and CD68-594 or CD11b-594 and representative images were pseudo-colored with red (Iba1) and green (CD68 and CD11b) based on personal preference (representative images displaying merged Iba1 and CD68; Supplemental Fig. S2).

Following Golgi-Cox staining, sections containing the dorsal hippocampus (−2.0 to −2.5 mm from Bregma) were imaged using a Zeiss Axioplan II fluorescence light microscope (Carl Zeiss, Thornwood, NY) with Cooke Sensicam cooled CCD. Five bright-field z-stack images of Golgi stained dendrites were captured per animal using a Zeiss Plan-Apo 63x/1,40 Oil objective (up to 80 μm total per z-stack at 0.5 μm steps).

### Microglial sholl analysis (Iba1)

Confocal acquired z-stacks were imported into Image-J and compressed into a max z-projection in which individual microglia whose process arbor was within the image border (8–12 microglia per image) were selected and cropped into a new, blank image. Images of individual microglia were thresholded to create binarized arbor outlines, despeckled once to remove artifacts, and analyzed using the semi-automated Image-J Sholl plugin^6,64^. Five animals per sex per group, four images per animal, and 8–12 microglia per image for a total of 40 microglia per animal were analyzed.

### Iba1, CD68, and CD11b IHC analysis

Confocal acquired z-stack images of the hippocampal molecular layer were imported into Image-J and compressed into a max z-projection (five animals per sex per group, four images per animal). These images were generated from tissue that was co-labeled with Iba1 and CD68 or CD11b. The entire upper blade of the molecular layer was traced and cropped, Iba1-postive microglia were counted, and images were thresholded (the minimum threshold value was based on the average of ten randomly selected images) and the number of positively labeled pixels over the image area was used to generate percent area covered for Iba1 (Supplemental Fig. S1), CD68, and CD11b (Fig. 2).

### Golgi Stain spine analysis

This analysis was replicated from the very detailed, step-by-step explanation published by Risher *et al*.^47^. A minimum of 50 μm was analyzed per dendrite broken into 10 to 50 μm segments with a set magnification of “27” across all images (27 refers to RECONSTRUCT’s magnification setting). Across five z-stacks, a total dendritic length of 500 μm was analyzed per animal according to Risher’s method with a total of four animals per sex per group. After tracing was complete, spine width and length numbers were copied into the “Spreadsheet S1” template^47^ and graphed according to spine density (protrusions per micron) and average spine type based on spine morphology (filopodia, long, thin, stubby, mushroom, and branched).

### Statistical analyses

Data were presented as mean ± SEM and all statistical analyses were carried out in Graphpad Prism 8.1 (GraphPad Software, La Jolla, California USA) to evaluate differences between sham-irradiated and irradiated, genotype, and sex differences. Microglial area under curve, max peak values, CD68 percent area, and Golgi spine density were analyzed by three-way ANOVA followed by Sidak multiple comparisons test (values displayed in Supplementary Fig. S4). Sholl analysis, percent area CD11b, and Golgi spine morphology were analyzed using two-way ANOVA with Holm-Sidak multiple comparisons test. P = 0.05 was considered significant (*p < 0.05, **p < 0.01, ***p < 0.001, ****p < 0.0001).

## Supplementary information

Supplemental Information

## Data Availability

The datasets generated and analyzed during the current study are available from the corresponding author on reasonable request.
