# Supplementary material for: Cranial irradiation mediated spine loss is sex-specific and complement receptor-3 dependent in male mice
Source: Sci Rep. 2019 Dec 11;9:18899. doi: 10.1038/s41598-019-55366-6 (PMC6906384; doi:10.1038/s41598-019-55366-6)
Supplement: Supplementary file 1 — Supplemental Information [file 41598_2019_55366_MOESM1_ESM.pdf]

Supplementary information

Cranial irradiation mediated spine loss is sex-specific and complement receptor-3 dependent in male mice

Joshua J. Hinkle[1], John A. Olschowka[1], Tanzy M. Love[2], Jacqueline P. Williams[3,4], and M. Kerry O'Banion*[1, 5]
[1]Department of Neuroscience and Del Monte Neuroscience Institute,
[2]Department of Biostatistics and Computational Biology,
[3]Department of Environmental Medicine, and
[4]Department of Neurology,
University of Rochester School of Medicine & Dentistry,
Rochester, New York, 14642.

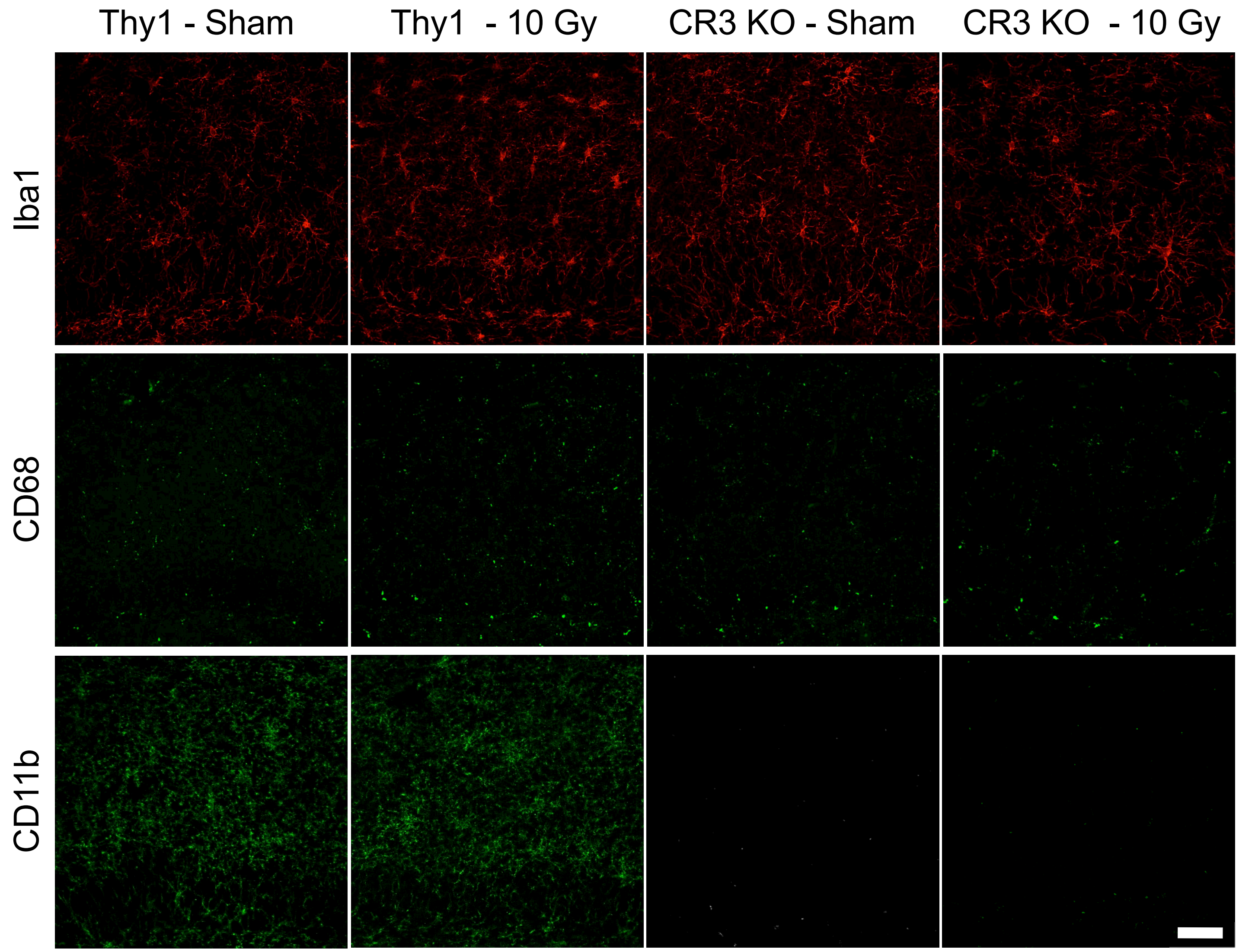

Figure S1. Representative confocal Iba1, CD68, and CD11b immunofluorescent max projection z-stack images from female Thy1+ and CR3 KO animals, either sham-irradiated or 10 Gy, taken in the hippocampal molecular layer. Scale bar: 50 µm.

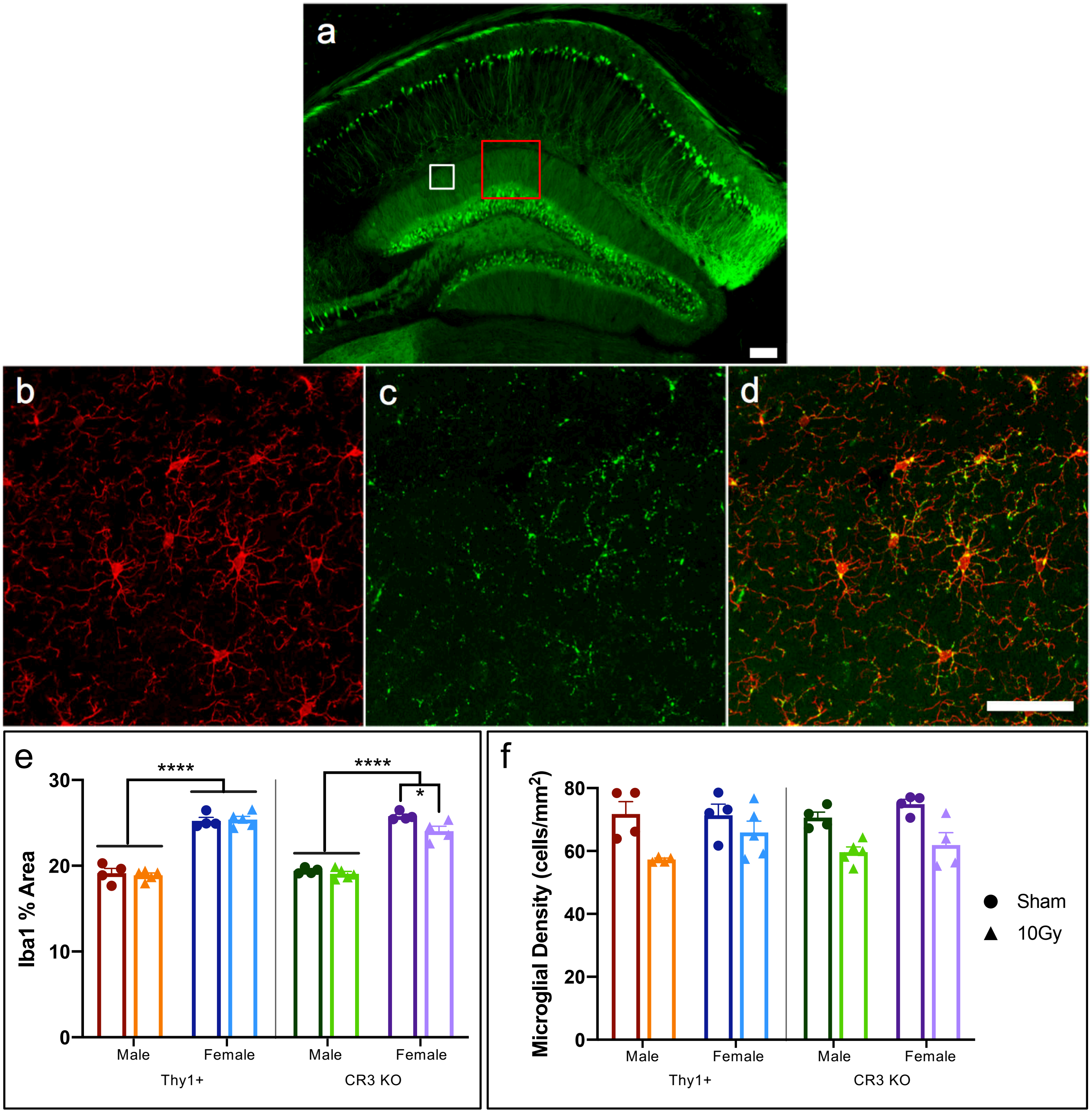

Figure S2. Representative confocal immunofluorescent max projection z-stack images of **a**) Thy1+ hippocampus and magnified molecular layer (white box) demonstrating **b**) Iba1, **c**) CD68, and **d**) Iba1 and CD68 merged image to display localization of Iba1 and CD68 (yellow). The red box (a) indicates the field size and region where 3-4 images were taken across the apical portion of the molecular layer for Iba1, CD68, and CD11b quantification. Quantification of **e**) Iba1 percent area immunofluorescence and **f**) microglial density demonstrating a significant increase in Iba1 area covered in female mice when compared to male mice and no significant difference is density (10Gy IR density reduction trends are non-significant). Scale bar: **a**) 100 µm; **b-d**) 50 µm. **e, f**) n = 3 sections averaged per animal, 4-5 animals per group; three-way ANOVA followed by Sidak's multiple comparisons; * $p < .05$, **** $p < 0.0001$.

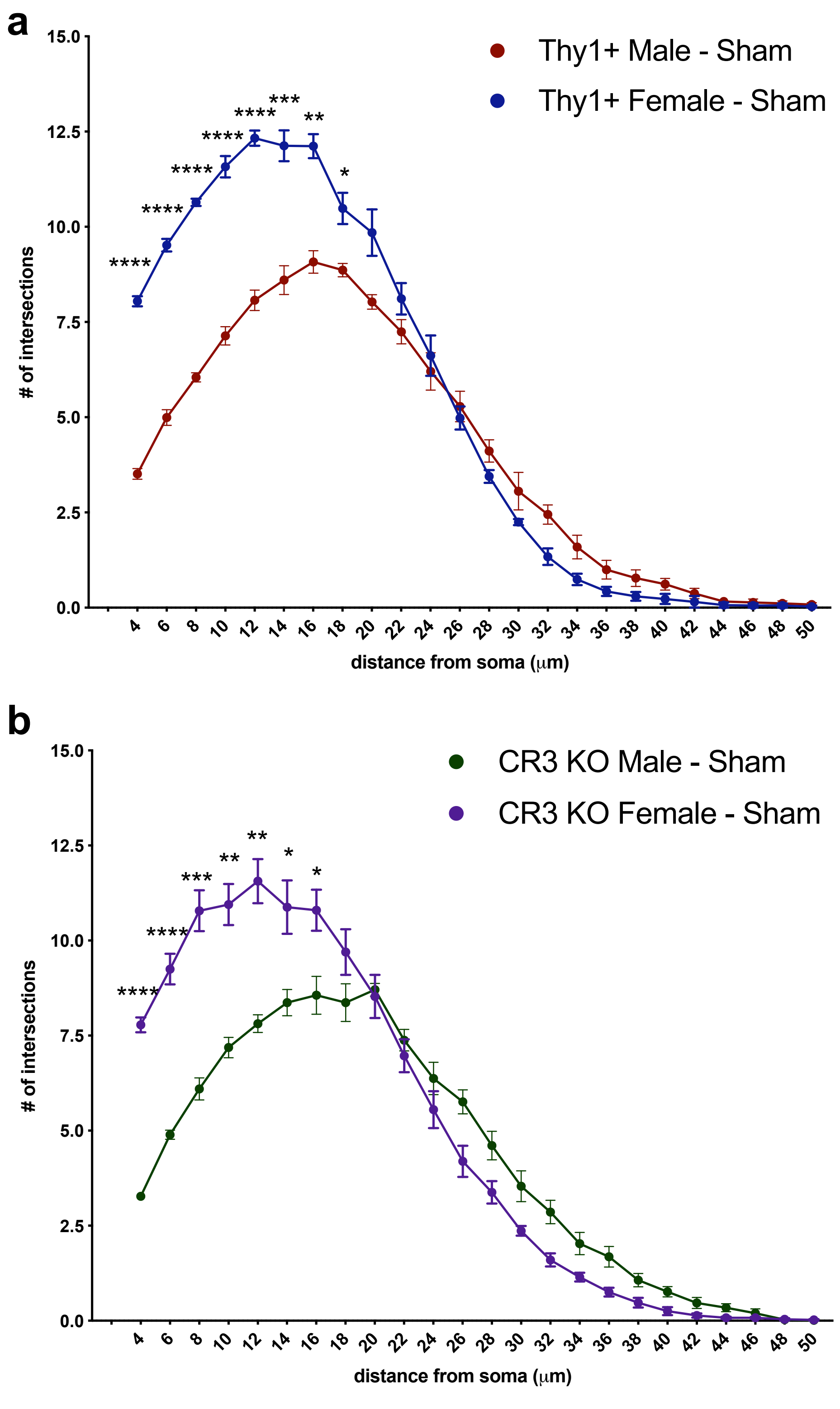

Figure S3. Morphological Sholl analysis of microglial arbor showing the significant basal sex difference in **a**) Thy1+ and **b**) CR3 KO animals. These two graphs were pulled directly from fig. 2 to better illustrate the significant difference between male and female sham-irradiated animals. n = 5 per group; **a, b**) two-way ANOVA followed by multiple t-tests with Holm-Sidak correction; * p< 0.05, ** p< 0.01, *** p<0.001, **** p<0.0001.

Three-way ANOVA:	A. Sholl Area Under Curve		B. Sholl Max Peak Value		C. CD68 % Area	
	$F_{(1, 32)}$ =	**P-value**	$F_{(1, 32)}$ =	**P-value**	$F_{(1, 32)}$ =	**P-value**
Sex	61.36	**<0.0001**	148.3	**<0.0001**	344.6	**<0.0001**
Genotype	0.0707	0.7917	8.215	**0.0073**	0.9579	0.3351
IR	28.58	**<0.0001**	6.012	**0.0198**	44.94	**<0.0001**
Sex x Genotype	1.319	0.299	3.486	0.0711	0.2077	0.6516
Sex x IR	1.593	0.2647	2.115	0.1556	13.02	**0.001**
Genotype x IR	0.3124	0.6292	0.6378	0.4304	0.8974	0.3506
Sex x Genotype x IR	0.4266	0.5621	0.4113	0.5259	0.07132	0.7911
Residual	**DF:** 32	**MS:** 93.94	**DF:** 32	**MS:** 0.734	**DF:** 32	**MS:** 0.019
**Sidak's Multiple Comparisons:**						
	**Diff. of Means**	**Adjusted p-value**	**Diff. of Means**	**Adjusted p-value**	**Diff. of Means**	**Adjusted p-value**
Thy1 Sham: Male vs. Female	-25.66	**0.0025**	-3.236	**<0.0001**	0.6588	**<0.0001**
Thy1 IR: Male vs. Female	-29.4	**0.0004**	-4.371	**<0.0001**	0.957	**<0.0001**
CR3 KO Sham: Male vs. Female	-14.62	0.2454	-2.572	**0.0005**	0.6756	**<0.0001**
CR3 KO IR: Male vs. female	-26.36	**0.0018**	-3.012	**<0.0001**	1.021	**<0.0001**
Thy1 Male: Sham vs. IR	-12.81	0.4226	-0.3128	>0.9999	-0.4905	**<0.0001**
CR3 KO Male: Sham vs. IR	-12.23	0.4905	-0.2276	>0.9999	-0.4298	**0.0004**
Thy1 Female: Sham vs. IR	-16.54	0.1247	-1.448	0.132	-0.1923	0.3782
CR3 KO Female: Sham vs. IR	-23.97	**0.0054**	-0.668	0.9541	-0.0839	0.9947
Male Sham: Thy1+ vs. CR3 KO	-2.994	>0.9999	0.228	>0.9999	-0.00702	>0.9999
Male IR: Thy1+ vs. CR3 KO	-2.416	>0.9999	0.3132	>0.9999	0.05368	>0.9999
Female Sham: Thy1+ vs. CR3 KO	8.05	0.9297	0.892	0.751	0.00982	>0.9999
Female IR: Thy1+ vs. CR3 KO	0.62	>0.9999	1.672	0.0488	0.1182	0.9257

Three-way ANOVA:	D. Golgi Spine Density		E. Iba1 % Area		F. Microglial Density	
	$F_{(1, 24)}$ =	**P-value**	$F_{(1, 27)}$ =	**P-value**	$F_{(1, 27)}$ =	**P-value**
Sex	37.61	**< 0.0001**	501.6	**<0.0001**	2.074	0.1613
Genotype	9.459	**0.0052**	0.1061	0.7471	0.05246	0.8206
IR	4.303	**0.0489**	4.115	0.0525	24.02	**<0.0001**
Sex x Genotype	15.32	**0.0007**	1.443	0.2401	0.01583	0.9008
Sex x IR	0.001	0.98	0.9053	0.3498	0.2664	0.61
Genotype x IR	25.25	**0.0001**	3.58	0.0693	0.6281	0.435
Sex x Genotype x IR	12.68	0.0016	2.873	0.1016	0.985	0.3298
Residual	**DF:** 24	**MS:** 0.023	**DF:** 27	**MS:** 0.617	**DF:** 27	**MS:** 38.31
**Sidak's Multiple Comparisons:**						
	**Diff. of Means**	**Adjusted p-value**	**Diff. of Means**	**Adjusted p-value**	**Diff. of Means**	**Adjusted p-value**
Thy1 Sham: Male vs. Female	-0.3458	**0.0395**	-6.105	**<0.0001**	0.4086	>0.9999
Thy1 IR: Male vs. Female	-0.7213	**<0.0001**	-6.502	**<0.0001**	-5.941	0.8379
CR3 KO Sham: Male vs. Female	-0.3083	0.0899	-6.369	**<0.0001**	-4.298	0.9925
CR3 KO IR: Male vs. female	0.07267	0.9998	-4.955	**<0.0001**	-2.293	>0.9999
Thy1 Male: Sham vs. IR	0.5648	**0.0002**	0.2351	>0.9999	11.82	0.0955
CR3 KO Male: Sham vs. IR	-0.3472	**0.0383**	0.3403	0.9999	10.98	0.1501
Thy1 Female: Sham vs. IR	0.1893	0.6663	-0.1622	>0.9999	5.471	0.9299
CR3 KO Female: Sham vs. IR	0.03376	>0.9999	1.754	**0.0457**	12.98	0.0722
Male Sham: Thy1+ vs. CR3 KO	0.0848	0.9989	-0.2864	>0.9999	1.168	>0.9999
Male IR: Thy1+ vs. CR3 KO	-0.8272	**<0.0001**	-0.1813	>0.9999	0.326	>0.9999
Female Sham: Thy1+ vs. CR3 KO	0.1223	0.9734	-0.5503	0.9919	-3.539	0.9987
Female IR: Thy1+ vs. CR3 KO	-0.03321	>0.9999	1.366	0.1679	3.974	0.994

Table S4. Thy1+ and CR3 KO Three-way ANOVA and Sidak's Multiple Comparisons data from **A**) Sholl Area Under Curve (Fig. 2b), **B**) Sholl Max Peak Value (Fig. 2c), **C**) CD68 % Area (Fig. 3a), **D**) Golgi Spine Density (Fig. 4c), **E**) Iba1 % Area (Fig. S1e), **F**) Microglial Density (Fig. S1f). Diff. of means is mean 1 – mean 2 and $p < .05$ values are displayed in bold.

	Thy1+				CR3 KO			
	Male		Female		Male		Female	
	Sham	IR	Sham	IR	Sham	IR	Sham	IR
**Area Under Curve**	95.74 ± 2.967	108.5 ± 3.431	121.4 ± 2.904	137.9 ± 8.097	98.73 ± 4.323	110.9 ± 1.918	113.4 ± 5.305	137.36 ± 2.287
**Max Peak Value**	9.288 ± 0.262	9.601 ± 0.427	12.52 ± 0.143	13.97 ± 0.639	9.060 ± 0.325	9.288 ± 0.239	11.63 ± 0.561	12.30 ± 0.130
**CD68 % area**	1.645 ± 0.037	2.135 ± 0.068	0.986 ± 0.062	1.178 ± 0.081	1.652 ± 0.048	2.081 ± 0.089	0.976 ± 0.061	1.065 ± 0.039
**CD11b % area**	17.55 ± 0.378	24.55 ± 0.608	12.04 ± 0.170	12.03 ± 0.367	----	----	----	----
**Spine density**	1.874 ± 0.056	1.310 ± 0.039	2.195 ± 0.883	2.03 ± 0.118	1.790 ± 0.068	2.137 ± 0.098	2.089 ± 0.051	2.064 ± 0.071
**Spine Morphology:**								
Filopodia	35.25 ± 5.543	11.25 ± 2.136	28.75 ± 6.408	29.50 ± 12.45	44.75 ± 3.224	33.50 ± 3.202	38.00 ± 6.519	42.25 ± 9.232
	t = 4.041, **p = 0.041**		t = 0.054, p = 0.995		t = 2.696, p = 0.196		t = 0.376, p = 0.993	
Long	450.0 ± 37.87	270.3 ± 27.81	481.6 ± 84.41	493.5 ± 27.81	347.3 ± 95.87	393.5 ± 62.79	452.5 ± 53.70	492.5 ± 82.78
	t = 3.826, **p = 0.042**		t = 0.132, p = 0.995		t = 0.404, p = 0.947		t = 0.405, p = 0.993	
Thin	273.3 ± 38.20	188.3 ± 21.32	229.8 ± 55.51	251.0 ± 34.22	202.8 ± 42.22	232.3 ± 24.55	249.0 ± 41.38	281.3 ± 48.12
	t = 1.943, p = 0.190		t = 0.326, p = 0.995		t = 0.604, p = 0.947		t = 0.508, p = 0.993	
Mushroom	130.3 ± 17.56	123.8 ± 27.05	215.0 ± 36.54	235.5 ± 44.48	289.3 ± 61.92	343.0 ± 58.63	263.3 ± 35.68	160.5 ± 33.54
	t = 0.202, p = 0.847		t = 0.356, p = 0.995		t = 0.630, p = 0.947		t = 2.098, p = 0.396	
Stubby	24.50 ± 4.213	8.000 ± 2.380	51.75 ± 13.54	74.75 ± 13.26	43.25 ± 9.259	51.25 ± 7.215	45.75 ± 8.957	46.50 ± 7.263
	t = 3.410, p = 0.056		t = 1.214, p = 0.849		t = 0.682, p = 0.947		t = 0.065, p = 0.997	
Branched	11.25 ± 0.250	12.00 ± 0.00	11.25 ± 1.125	10.50 ± 0.645	13.25 ± 1.702	10.00 ± 1.581	11.50 ± 1.555	11.50 ± 1.708
	t = 3.000, p = 0.070		t = 0.553, p = 0.991		t = 1.399, p = 0.695		t = 0.0, p > 0.999	

Table S5. Mean ± SEM values across Thy1+ and CR3 KO groups listed for Area Under Curve, Max Peak Value, CD68 % Area, CD11b % Area, spine density, and spine morphology. Spine morphology displays six spine types followed by t- and p-values from Multiple t-tests with Holm-Sidak correction.

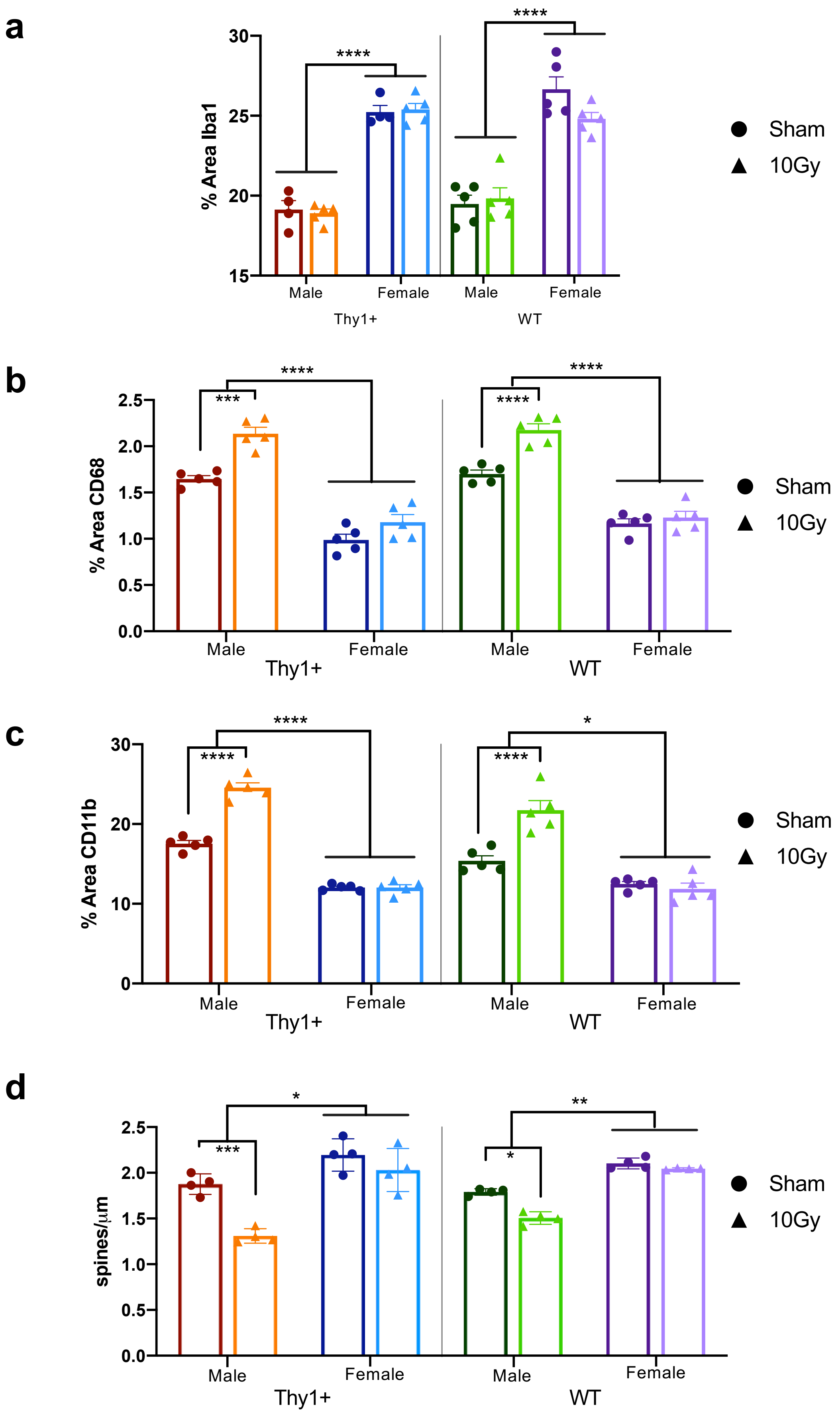

Figure S6. Quantification of Thy1+ and WT a) Iba1 % area, **b**) CD68 % area, **c**) CD11b % area, and **d**) spine density. Thy1+ and WT animals exhibited similar mean values and trends with no significant differences demonstrating that the quantification of Thy1+ and WT markers respond similarly to radiation. **a-c**) n = 5 per group, **d**) n=4 per group; **a-d**) 3-way ANOVA with multiple comparisons; * p< 0.05, ** p< 0.01, ** p< 0.001, **** p< 0.0001.

Three-way ANOVA:	A. Iba1 % Area		B. CD68 % Area		C. CD11b % Area	
	$F_{(1, 32)}$ =	P-value	$F_{(1, 32)}$ =	P-value	$F_{(1, 32)}$ =	P-value
Sex	267.5	**<0.0001**	328.9	**<0.0001**	301.7	**<0.0001**
Genotype	1.958	0.172	3.579	0.0676	7.011	**0.0125**
IR	1.087	0.3056	51.16	**<0.0001**	51.29	**<0.0001**
Sex x Genotype	0.1004	0.7535	0.6309	0.4329	8.766	**0.0057**
Sex x IR	1.449	0.2381	17.19	**0.0002**	61.81	**<0.0001**
Genotype x IR	0.8948	0.3517	0.7149	0.4041	0.5319	0.4711
Sex x Genotype x IR	2.99	0.094	0.4266	0.5183	0.0007724	0.978
Residual	**DF:** 32	**MS:** 1.345	**DF:** 32	**MS:** 0.0182	**DF:** 32	**MS:** 1.969
**Sidak's Multiple Comparisons:**						
	**Diff. of Means**	**Adjusted p-value**	**Diff. of Means**	**Adjusted p-value**	**Diff. of Means**	**Adjusted p-value**
Thy1 Sham: Male vs. Female	-6.105	**<0.0001**	0.6588	**<0.0001**	5.52	**<0.0001**
Thy1 IR: Male vs. Female	-6.502	**<0.0001**	0.957	**<0.0001**	12.52	**<0.0001**
WT Sham: Male vs. Female	-7.173	**<0.0001**	0.5352	**<0.0001**	2.917	**0.0291**
WT IR: Male vs. female	-4.955	**<0.0001**	0.9449	**<0.0001**	9.869	**<0.0001**
Thy1 Male: Sham vs. IR	0.2351	>0.9999	-0.4905	**<0.0001**	-7.002	**<0.0001**
WT Male: Sham vs. IR	-0.3572	>0.9999	-0.474	**<0.0001**	-6.33	**<0.0001**
Thy1 Female: Sham vs. IR	-0.1622	>0.9999	-0.1923	0.3172	-0.0006	>0.9999
WT Female: Sham vs. IR	1.861	0.1824	-0.06433	0.9993	0.6219	0.9997
Male Sham: Thy1+ vs. WT	-0.3527	>0.9999	-0.05505	0.9999	2.153	0.2257
Male IR: Thy1+ vs. WT	-0.945	0.9387	-0.03864	>0.9999	2.824	**0.0382**
Female Sham: Thy1+ vs. WT	-1.421	0.6219	-0.1786	0.4203	-0.4501	>0.9999
Female IR: Thy1+ vs. WT	0.6022	0.9985	-0.05068	>0.9999	0.1724	>0.9999

Three-way ANOVA:	D. Golgi Spine Density	
	$F_{(1, 24)}$ =	P-value
Sex	143.6	**<0.0001**
Genotype	0.002352	0.9617
IR	46.89	**<0.0001**
Sex x Genotype	1.805	0.1917
Sex x IR	14.17	**0.001**
Genotype x IR	6.604	**0.0168**
Sex x Genotype x IR	0.858	0.3635
Residual	**DF:** 24	**MS:** 0.0128
**Sidak's Multiple Comparisons:**		
	**Diff. of Means**	**Adjusted p-value**
Thy1 Sham: Male vs. Female	-0.3458	**0.0028**
Thy1 IR: Male vs. Female	-0.7213	**<0.0001**
WT Sham: Male vs. Female	-0.3124	**0.0081**
WT IR: Male vs. female	-0.5396	**<0.0001**
Thy1 Male: Sham vs. IR	0.5648	**<0.0001**
WT Male: Sham vs. IR	0.2849	**0.019**
Thy1 Female: Sham vs. IR	0.1893	0.2758
WT Female: Sham vs. IR	0.05767	0.9996
Male Sham: Thy1+ vs. WT	0.08424	0.9869
Male IR: Thy1+ vs. WT	-0.1957	0.2366
Female Sham: Thy1+ vs. WT	0.1176	0.867
Female IR: Thy1+ vs. WT	-0.01395	>0.9999

Table S7. Thy1+ and WT KO Three-way ANOVA and Sidak's Multiple Comparisons data from **A**) Iba1 % area, **B**) CD68 % area, **C**) CD11b % area, and **D**) spine density. Diff. of means is mean 1 – mean 2 and $p < .05$ values are displayed in bold.
